# Supplementary material for: Persistence of vestibular function in the absence of glutamatergic transmission from hair cells
Source: Sci Rep. 2026 Mar 23;16:14550. doi: 10.1038/s41598-026-43836-7 (PMC13153426; doi:10.1038/s41598-026-43836-7)
Supplement: Supplementary file 1 — Supplementary Material 1 [file 41598_2026_43836_MOESM1_ESM.pdf]

Supplementary Information for

Persistence of vestibular function in the absence of glutamatergic transmission from hair cells

Mohona Mukhopadhyay[1], Ruchi Modgekar[1,2], Aizhen Yang-Hood[3], Kevin K. Ohlemiller[3], Valentin Militchin[3], Maolei Xiao[3], Zhijun Shen[3], Nicholas R Rensing[4], Michael Wong[4], Suh Jin Lee[5], Rebecca P. Seal[5], Joseph C. Holt[6], Mark E. Warchol[3], Susan E. Maloney[7,8], Carla M. Yuede[7,8], Mark A. Rutherford[3*], Tina Pangrsic[1,9,10,11*]

[1]Experimental Otology Group, InnerEarLab, Department of Otolaryngology, University Medical Center Göttingen, and Institute for Auditory Neuroscience, Göttingen, Germany

[2]International Max Planck Research School (IMPRS) for Neuroscience, Göttingen, Germany

[3]Department of Otolaryngology – Head and Neck Surgery, Washington University School of Medicine, St. Louis, MO, USA

[4]Department of Neurology, Washington University School of Medicine, St. Louis, MO, USA

[5]Departments of Neurobiology and Otolaryngology, Pittsburgh Center for Pain Research, University of Pittsburgh, Pittsburgh, PA, USA

[6]Department of Otolaryngology and Department of Neuroscience, University of Rochester, Rochester, NY, USA

[7]Department of Psychiatry, Washington University School of Medicine, St. Louis, MO, USA

[8]Intellectual and Developmental Disability Research Center, Washington University School of Medicine, St. Louis, MO, USA

[9]Institute for Auditory Neuroscience, Göttingen, Germany, Auditory Neuroscience Group, Max Planck Institute for Multidisciplinary Sciences, Göttingen, Germany

[10]Collaborative Research Center 889, University of Göttingen, Göttingen, Germany

[11]Multiscale Bioimaging Cluster of Excellence (MBExC), University of Göttingen, Göttingen, Germany.

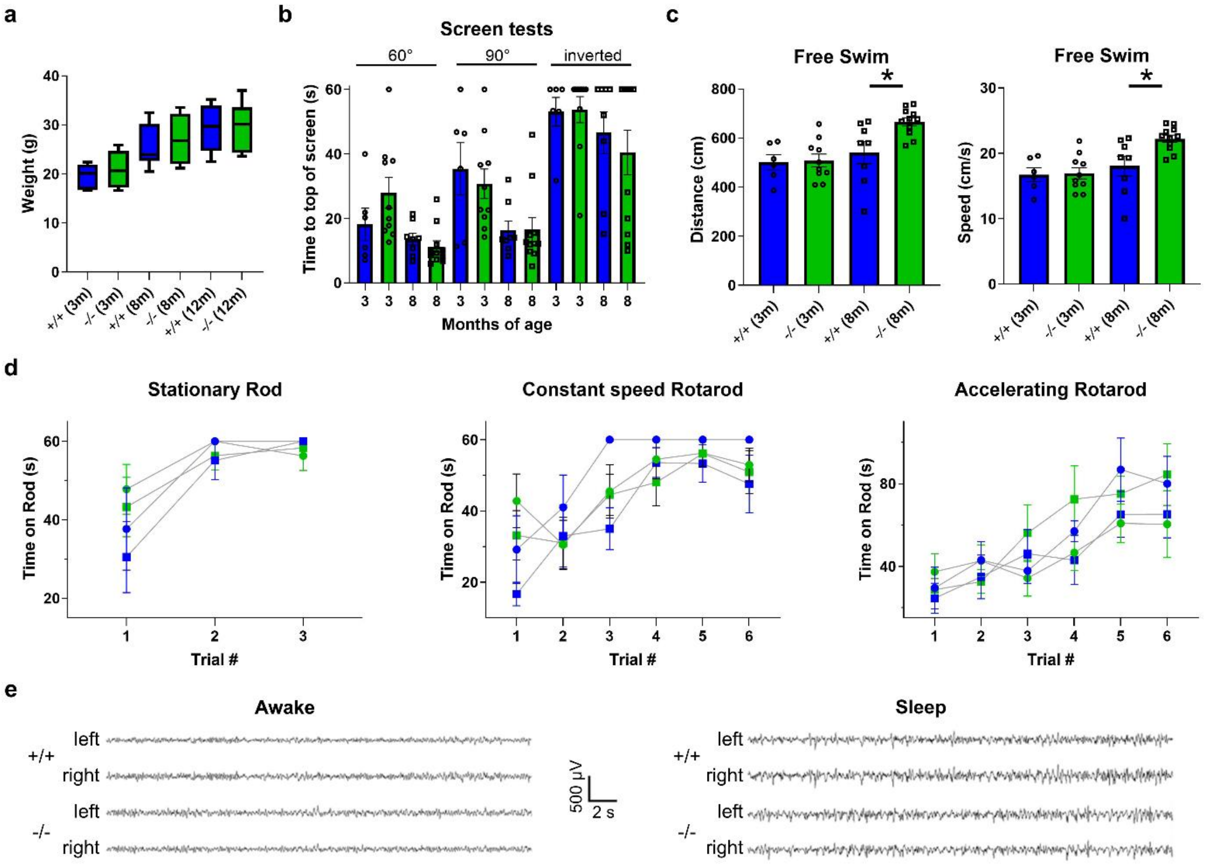

Supplemental Figure 1. Climbing, rotarod and swimming performance in the presence or absence of VGLUT3.

a) Body weight was similar between *Vglut3*$^{+/+}$ and *Vglut3*$^{-/-}$ mice. **b)** In the three screen climbing tests, mice of each genotype performed similarly at 3 and 8 months ($p > 0.05$, Mann Whitney U test). **c)** At 8 months, the *Vglut3*$^{-/-}$ mice swam further and faster ($p = 0.01$, Mann Whitney U test). **d)** In the three rod tests, mice of each genotype performed similarly at 3 and 8 months ($p > 0.05$, Mann Whitney U test). N = 6 WT, 10 KO at 3 months; 8 WT and 11 KO mice at 8 and 12 months. **e)** Representative EEG tracings from *Vglut3*$^{-/-}$ (N = 5) and control mice (N = 3) during the awake (*left*) and sleep (*right*, non-REM) states.

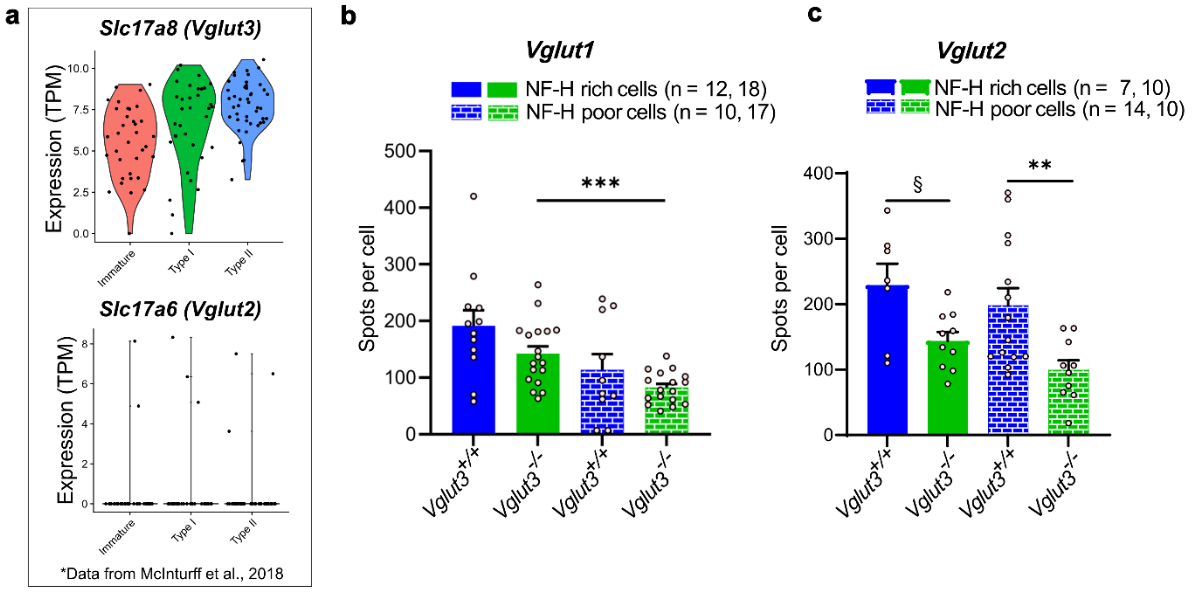

Supplemental Figure 2. *Vglut1*, *2* and *3* transcripts in the vestibular hair cells and vestibular ganglion.

a) VGLUT3 (encoded by *Slc17a8* gene; *top*) and VGLUT2 (encoded by *Slc17a6* gene; *bottom*) expression in immature VHCs, mature type I VHCs, and mature type II VHCs (single cell RNA sequencing extracted from ref. [41]). Data publicly available at umgear.org. TPM units stand for transcript per million. **b)** Abundance of *Vglut1* mRNA spots in the NF-H/TuJ1-rich vs NF-H/TuJ1-poor neurons of both genotypes. **c)** Abundance of *Vglut2* mRNA spots in the NF-H/TuJ1-rich vs NF-H/TuJ1-poor neurons of both genotypes. Student's *t* test and Holm-Bonferroni correction for multiple comparison; $p = 0.0004$ (asterisk in b); $p = 0.0075$ and 0.017 (asterisk and § in c, respectively).

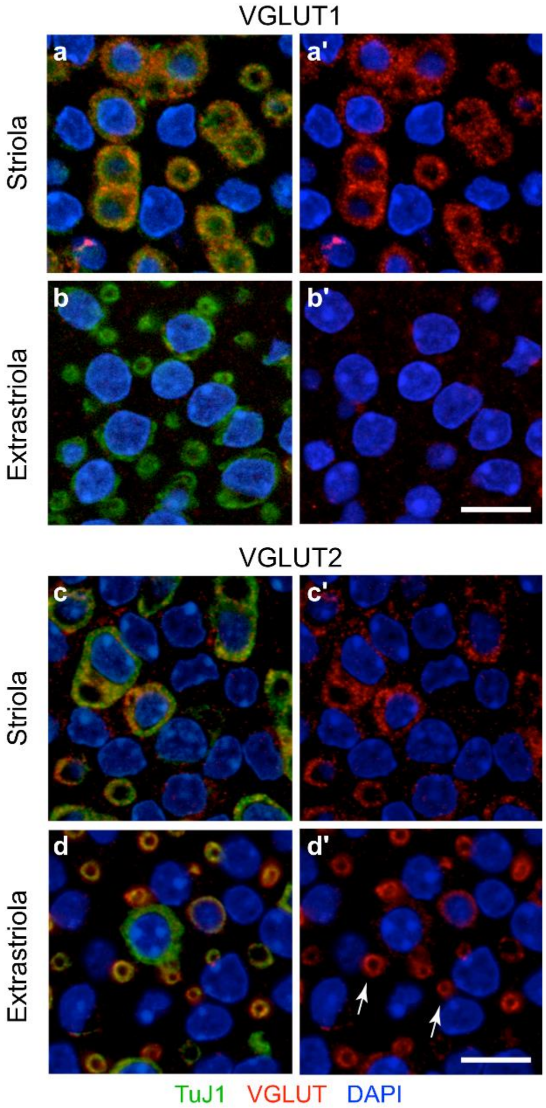

Supplemental Figure 3. Immunoreactivity for VGLUT1 and VGLUT2 in the mouse utricle. a,a') Labeling for VGLUT1 was observed in all calyx terminals in the striola. **b,b')** VGLUT1 immunoreactivity was very weak (or absent) in the extra-striolar regions. **c,c')** VGLUT2 immunoreactivity was observed in some striolar calyces. **d,d')** VGLUT2 labeling was observed in all calyces in the extra-striolar regions. Labeling was particulary strong in the 'neck' region of the calyx (*arrows*). Scale bars: 10 µm.

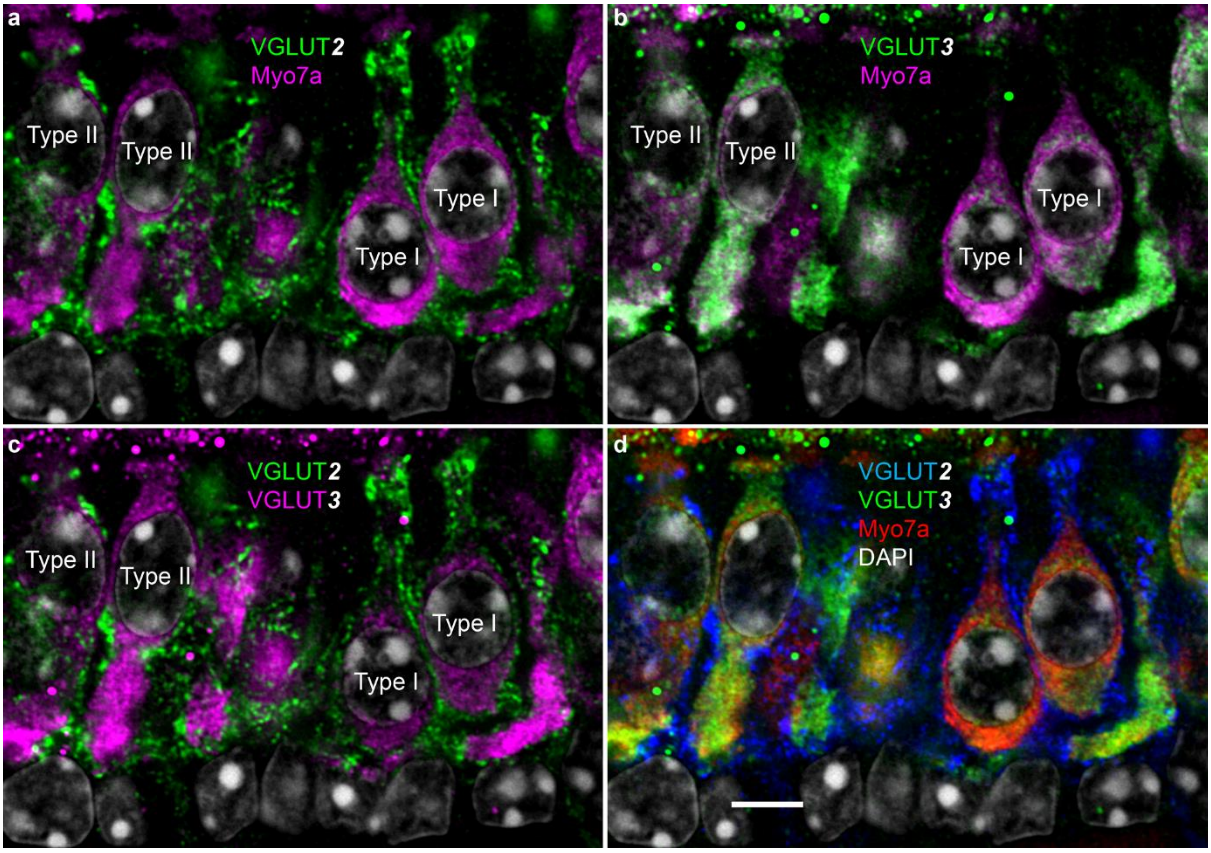

Supplemental Figure 4. Further examples of VGLUT2 and VGLUT3 expression profile in the *Vglut3*$^{+/+}$ mouse utricle.

Single optical sections of Airyscan microscopy on an exemplary utricular cryosection in the central zone from an 8-week old mouse labelled with DAPI, Myosin7a, VGLUT2, and VGLUT3. **a)** The VHCs express Myosin7a (*magenta*). VGLUT2 (*green*) robustly labels the space surrounding the necks of type I VHCs, as well as below and around the basolateral hair cell membranes. **b)** In contrast, VGLUT3 (*green*) robustly labels the type II VHC cytoplasm and weakly labels the type I VHC cytoplasm. **c)** In the same field of view, VGLUT2 (*green*) and VGLUT3 (*magenta*) are predominantly non-overlapping. **d)** All four markers in the same field of view: VGLUT2 (*blue*), VGLUT3 (*green*), Myosin7a (*red*), DAPI (*white*). Scale bar: 5 µm.

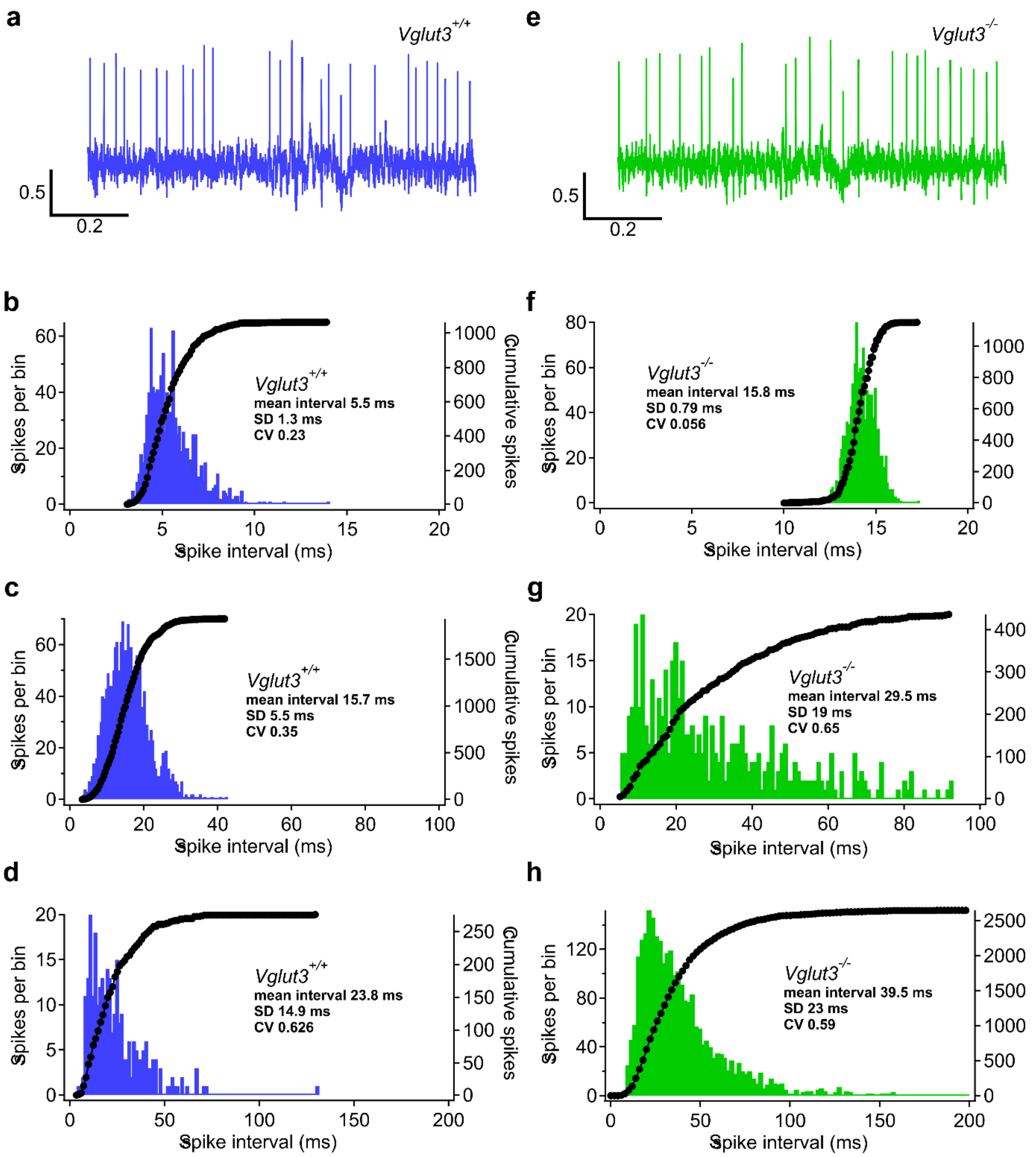

Supplemental Figure 5. Exemplary spontaneous spiking in the vestibular neurons of mice of both genotypes.
a) Example recordings of spontaneous activity in the vestibular nerve from *Vglut3*$^{+/+}$ mouse. **b-d)** Characterization of three vestibular units from *Vglut3*$^{+/+}$ mouse with binned and cumulative spike histograms. **e)** As in a but from a *Vglut3*$^{-/-}$ mouse. **f-h)** As in b-d but from a *Vglut3*$^{-/-}$ mouse.
